# Effects of over-expressing a native gene encoding 5-enolpyruvylshikimate-3-phosphate synthase (EPSPS) on glyphosate resistance in *Arabidopsis thaliana*

**DOI:** 10.1371/journal.pone.0175820

**Published:** 2017-04-20

**Authors:** Xiao Yang, Zachery T. Beres, Lin Jin, Jason T. Parrish, Wanying Zhao, David Mackey, Allison A. Snow

**Affiliations:** 1 Department of Evolution, Ecology, and Organismal Biology, Ohio State University, Columbus, Ohio, United States of America; 2 Department of Horticulture and Crop Science, Ohio State University, Columbus, Ohio, United States of America; National Taiwan University, TAIWAN

## Abstract

Widespread overuse of the herbicide glyphosate, the active ingredient in RoundUp^®^, has led to the evolution of glyphosate-resistant weed biotypes, some of which persist by overproducing the herbicide’s target enzyme, 5-enolpyruvylshikimate-3-phosphate synthase (EPSPS). EPSPS is a key enzyme in the shikimic acid pathway for biosynthesis of aromatic amino acids, lignin, and defensive compounds, but little is known about how overproducing EPSPS affects downstream metabolites, growth, or lifetime fitness in the absence of glyphosate. We are using *Arabidopsis* as a model system for investigating phenotypic effects of overproducing EPSPS, thereby avoiding confounding effects of genetic background or other mechanisms of herbicide resistance in agricultural weeds. Here, we report results from the first stage of this project. We designed a binary vector expressing a native *EPSPS* gene from *Arabidopsis* under control of the CaMV35S promoter (labelled OX, for over-expression). For both OX and the empty vector (labelled EV), we obtained nine independent T3 lines. Subsets of these lines were used to characterize glyphosate resistance in greenhouse experiments. Seven of the nine OX lines exhibited enhanced glyphosate resistance when compared to EV and wild-type control lines, and one of these was discarded due to severe deformities. The remaining six OX lines exhibited enhanced *EPSPS* gene expression and glyphosate resistance compared to controls. Glyphosate resistance was correlated with the degree of *EPSPS* over-expression for both vegetative and flowering plants, indicating that glyphosate resistance can be used as a surrogate for *EPSPS* expression levels in this system. These findings set the stage for examination of the effects of *EPSPS* over-expression on fitness-related traits in the absence of glyphosate. We invite other investigators to contact us if they wish to study gene expression, downstream metabolic effects, and other questions with these particular lines.

## Introduction

EPSPS (5-enolpyruvoylshikimate-3-phosphate synthase, EC2.5.1.19) is a key enzyme in the shikimic acid pathway, which accounts for more than ~30% of carbon fixed by photosynthesis in vascular plants [1,2]. This pathway, found in nearly all plants, bacteria, and fungi, produces growth hormones, aromatic amino acids, lignin, flavonoids, phenolics, salicylic acid, and other secondary metabolites involved in plant defense [3,4,5]. Glyphosate (N-(phosphonomethyl) glycine), the active ingredient in RoundUp^®^ (Monsanto Company), is a systemic herbicide that targets the shikimic acid pathway by inhibiting EPSPS (e.g., [6]). Today, millions of hectares of farmland are planted with transgenic, glyphosate-tolerant crops [7]. Most of these crop varieties have an *EPSPS* gene from *Agrobacteria* sp. strain CP4 that encodes an enzyme with greatly reduced sensitivity to inhibition by glyphosate [6].

Due to heavy reliance on glyphosate worldwide, more than 35 weed species have evolved resistance to this herbicide through a variety of mechanisms (e.g., [8,9]). For several of these weed species, glyphosate resistance is conferred by overproduction of EPSPS (e.g., [10,11]). Overproduction of EPSPS can occur due to mutations that result in extra copies of the *EPSPS* gene, as reported in *Amaranthus palmeri*, *A*. *tuberculatus*, *A*. *spinosus*, *Lolium multiflorum*, *Kochia scoparia*, *Eleusine indica*, and *Bromus diandrus* [8,12,13]. In *A*. *palmeri* from Georgia, glyphosate-resistant biotypes had ~40–100 copies of the *EPSPS* gene scattered on different chromosomes [14,15,16]. Fewer copies of the gene have been reported in other resistant biotypes. For example, *A*. *palmeri* from New Mexico had only 2–10 copies of the *EPSPS* gene [17], and *Kochia scoparia* from Colorado and Kansas had 3–9 tandem copies that exhibited Mendelian inheritance [18,19]. Overproduction of EPSPS also has been reported for some glyphosate-resistant biotypes of *Conyza canadensis* [20,21], although gene amplification has not been identified as the cause. Similarly, overproduction of EPSPS was reported for cell lines of carrot, tobacco, and petunia after many cycles of glyphosate-induced selection pressure in the laboratory [3,22,23].

While the benefit of overproduction of EPSPS in the presence of glyphosate is apparent, questions about whether overproduction affects plant survival, growth, and fecundity in the absence of exposure to glyphosate remain. For glyphosate-resistant weed biotypes cited in the preceding paragraph, EPSPS is overproduced in non-herbicide-treated plants and therefore may represent a constitutive difference in the plants’ metabolism. If the consequence of *EPSPS* over-expression is neutral or beneficial in terms of lifetime fitness in weed populations, and if the genetic basis for over-expression is stable over time, then it is possible that phenotypic traits associated with overproducing EPSPS could persist indefinitely. In contrast, if this mechanism for glyphosate resistance has a fitness cost under field conditions, then the underlying genetic trait is expected to be lost from weed populations after exposure to glyphosate is discontinued (e.g., [24,25]).

Most previous studies of plants that overproduce EPSPS have focused only on glyphosate resistance, without considering other phenotypic effects resulting from overproduction (reviewed in [26]). In a few cases, however, researchers tested for correlations between *EPSPS* copy number and fitness-related traits in weed species and found either no effect [27,28] or a possible negative correlation ([29]; EPSPS levels not published). A very different result emerged from our previous study of crop-weed hybrids from transgenic rice [26]. In this system, transgenic over-expression of a native *EPSPS* gene from cultivated rice was associated with increased seed germination, tryptophan concentrations, photosynthetic rates, and seed production in F2 and F3 crop-weed hybrids from several weedy accessions [26]. However, it is difficult to generalize from Wang et al. [26] because our experimental design involved a single transgenic event rather than multiple, independent transgenic lines, and therefore it is not possible to exclude effects of the transgene insertion or linked genes [30,31].

The overarching goal of our current research is to test for phenotypic effects of overproducing EPSPS in multiple lines of transgenic *Arabidopsis thaliana*. Brotherton et al. [32] noted that *Arabidopsis* is highly sensitive to glyphosate, and the availability of resistant lines would be useful for studying mechanisms of herbicide resistance. As recommended by the Weed Science Society of America, we use the term “resistance” to designate the emergence of heritable resistance relative to wild-type plants, rather than the term “tolerance”, which refers to a natural, inherent ability to withstand herbicide treatment [33]. This model system allows us to isolate effects of overproducing EPSPS from other possible confounding factors that can occur in glyphosate-resistant weed biotypes, such as different genetic backgrounds and multiple mechanisms and types of herbicide resistance (e.g., [34,35]). Our initial approach was similar to that of Klee et al. [36], who cloned an *EPSPS* gene from *Arabidopsis thaliana*, fused it to the CaMV35S promoter, and reintroduced the gene back into *Arabidopsis*. These authors showed that over-expressing *Arabidopsis* lines had superior callus growth and seedling survival compared to control lines when exposed to glyphosate, but they did not report data for mature plants, nor did they examine other phenotypic traits. In the present study, we created multiple transgenic lines that are homozygous for an over-expression construct (OX lines), along with control lines that are homozygous for an empty-vector construct (EV lines). Our specific questions in this first stage of the project were:

1. To what extent does glyphosate resistance vary among independent lines that share the OX construct *vs*. the EV construct?
2. Is greater resistance to glyphosate correlated with enhanced *EPSPS* gene expression, as expected?
3. Are differences in glyphosate resistance among lines consistent for both vegetative and reproductive plants?
4. Are differences in glyphosate resistance among lines repeatable in different experiments?

Briefly, we identified OX lines with enhanced glyphosate resistance and expected increases in *EPSPS* gene expression when compared to EV and wild-type control lines. Our results indicate that OX lines are more resistant to glyphosate in both vegetative and flowering plants across multiple experiments and that resistance correlates with the level of *EPSPS* over-expression. In a forthcoming paper, we will report variation in fitness-related traits among these OX, EV, and wild-type lines.

We invite other investigators to contact us if they wish to study additional aspects of gene expression, downstream metabolic effects, and other questions with these particular lines.

## Materials and methods

### Development of over-expressed and empty vector lines

**Fig 1:**
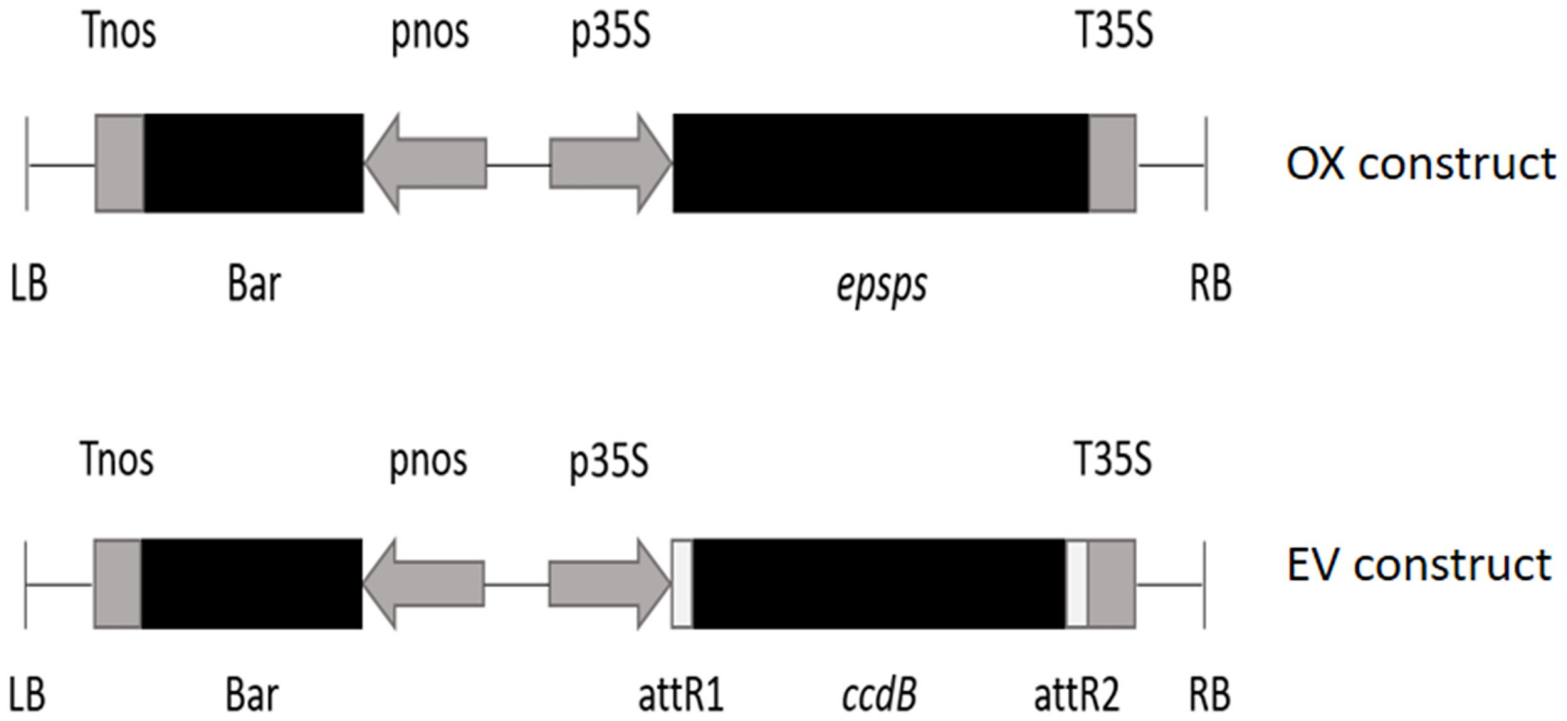
Diagrams of the OX (*EPSPS* over-expression) and EV (empty vector, pB2WG7) constructs. In the OX construct, the entry clone-*EPSPS* cDNA sequence was inserted between the attR1 and attR2 sites.

We developed *Arabidopsis* lines with independent insertions of over-expressed (OX) and empty vector (EV) constructs. *Arabidopsis* has two native *EPSPS* loci (AT1G48860 and AT2G45300 [14]) that are highly expressed throughout development (http://bar.utoronto.ca/efp/cgi-bin/efpWeb.cgi). We chose AT1G48860 due to its purported higher glyphosate resistance than AT2G45300 in *Arabidopsis* [37] and the G-clone (G09183, a gateway entry clone) was ordered from the Arabidopsis Biological Resource Center (ABRC) at Ohio State University. The entry clone was introduced into the binary expression vector pB2WG7 carrying the *Bar* gene conferring resistance to Basta (glufosinate) by LR recombination to generate the *EPSPS* construct, under the control of CaMV35S promoter (Fig 1). The *CcdB* (*control of cell death B*) gene from *Escherichia coli* present in the empty vector (EV) construct has no known function in plants. The OX construct was confirmed by sequencing, and both constructs were introduced to *Agrobacterium tumefaciens* strain GV3101. Then, GV3101 bearing either CaMV35S::*EPSPS* or the empty vector (Fig 1) was used to transform *Arabidopsis thaliana* (Columbia ecotype, Col-0) by the floral dip method [38].

Transgenic progeny were selected by spraying soil-grown seedlings on 3 consecutive days with 0.04% (w/v) Basta supplemented with 0.005% SilwetL-77 solution. Independent T1 lines with presumed single insertion loci were identified by 3:1 segregation of Basta resistance in the T2 generation. These T2 lines were allowed to self and homozygous T3 lines (those that segregated 4:0) were used in subsequent experiments. Using this procedure, 9 independent OX lines and 9 independent EV lines were generated. We also created OX and EV lines in a *sid2* (*eds16*, *ics1*) mutant of *Arabidopsis* that has reduced levels of defense-associated salicylic acid [39]. These *sid2* OX lines with enhanced glyphosate resistance and comparable empty vector lines also are available for study on request, but are not included in the current study.

### Glyphosate resistance

#### Dose-response experiment

To compare glyphosate resistance among the OX, EV, and wild-type, we conducted four independent experiments (Table 1). In February 2015, we began a dose-response experiment using 9 empty vector lines (EV), 9 over-expressed lines (OX), and the wild-type line. The experimental design included nine dosage treatments with three plants per line in each treatment, for a total of 513 plants. Seeds were germinated in the Biological Sciences Greenhouse at Ohio State University, Columbus, Ohio, in plastic flats with moistened Sun Gro Metro-Mix 360 (www.sungro.com). Each flat was divided into evenly spaced rows and lines were randomized within this grid, with one plant per line in each flat. Approximately 3–4 seeds were planted on the soil surface in each position and hand-watered using a fine mist. Upon germination, seedlings were thinned to one plant per position. The flats were rotated around the greenhouse weekly to minimize environmental variation and were hand-watered twice daily as needed. The greenhouse was maintained at 18-21/23-26 C (night/day) and supplemental lights (400-watt metal halide) were used for 14 hours per day. To maintain soil fertility, the flats were sprayed weekly with nutrient solution (180 ppm 20:10:20; www.jrpeters.com), starting two weeks after seedling emergence.

**Table 1:** Summary of greenhouse experiments to compare glyphosate resistance of OX, EV, and wild-type lines of *Arabidopsis thaliana*^1^.

Expmt ID	Numbers of plant lines	Date started	Final number of replicates^2.^	Mean rosette diameter (cm) prior to spraying^3.^	Percent flowering when sprayed^3.^	Time of spraying with glyphosate	Glyphosate dosages
**Dose Response**	9 OX, 9 EV, 1 wild-type	February 2015	3	5.8 (at 29 days)	~50%	35 days	0X through 16X (9 dosages)
**EXP A**	6 OX, 5 EV, 1 wild-type	June 2015	20	8.2 (at 43 days)	0	44 days	1.0X
**EXP B**	6 OX, 7 EV, 1 wild-type	November 2015	11–15	12.7 (at 40 days)	0	44 days	0.5X
**EXP C**^4.^	6 OX, 4 EV, 1 wild-type	March 2016	24	(no data)	~100%	35 days	0.5X

^1.^ OX (*EPSPS* over-expression) and EV (empty vector, pB2WG7) constructs.

^2.^ Number of plants per line in each glyphosate dosage treatment.

^3.^ Combined data from all plants and lines in the experiment to characterize average size and stage of development.

^4.^ These 6 OX lines and 3 of the 4 EV lines were used to quantify *EPSPS* gene expression.

At 35 days after planting, when the plants were ~5–7 cm in diameter, each tray was sprayed with one of 9 treatments: 0x, 0.001x, 0.004x, 0.0156x, 0.0625x, 0.25x, 1x, 4x, and 16x, where 1x is the recommended application rate for many agricultural weeds, 840 g ae/ha, and equates to 0.6725% glyphosate (v/v; AquaMaster^®^, 648 g L^-1^, Monsanto Co.; St. Louis, Missouri). Each treatment also contained ammonium sulfate solution (N-Pak^®^ AMS Liquid, 407 g L^−1^; Winfield Solutions, LLC; St. Paul, Minnesota) and non-ionic surfactant (Preference^®^; Winfield Solutions, LLC) at 5% and 0.5% (v/v), respectively. Treatments were applied using a pneumatic track sprayer equipped with an even, flat-spray tip (Teejet 8001EVS; Spraying Systems Co.; Carol Stream, Illinois) calibrated to apply 140 L ha^−1^ of spray solution at a speed of 3.5 km hr^−1^. Approximately half of the plants had started bolting and flowering at the time of spraying. At 14 days after treatment, the plants were visually assessed for damage on a scale from 0 (no damage) to 10 (dead). By 21 days, the plants showed signs of drought stress so they were not used for scoring resistance.

To quantify relative differences in glyphosate resistance among lines, we calculated the effective dose 50 (ED50; as in [40,41]). This dose corresponds to the midpoint between the lower and upper limits of the observed plant health. Using the *drc* package in R [40], the ED50 of each line was estimated using the four-parameter log-logistic function given in Eq 1:

$$Y=c+\left\{\frac{d-c}{1}+\text{exp}\left[b\left(logx-loge\right)\right]\right\}$$ (1)

where e is the ED50, the lower limit is c, the upper limit is d, and the parameter b denotes the relative slope around e. Our model constrained the lower limit to 0 (zero) and the upper limit to 10, which effectively reduced to the three-parameter log-logistic function. To avoid a statistical lack of fit to the model, data from the three lowest doses (0x, 0.001x, 0.004x) were designated as a zero dose. The selectivity index was calculated to allow statistical comparisons for ED50 estimates among lines at p<0.05 (Table 2).

**Table 2:** Relative resistance to glyphosate among 9 OX lines, 9 EV lines, and the wild-type line based on ED50 (kg ae/ha) estimates. ED50 (kg ae/ha) is the midpoint dosage of the dose-response curve for damage scores from zero (no damage) to 10 (dead), two weeks after spraying. The standard error (SE) is based on data from three plants for each of nine concentrations of glyphosate. Groups that do not share letters are significantly different at p<0.05.

Line ID	ED50 (kg ae/ha)	SE	Group
**OX1**	1.41	0.40	b
**OX2**	1.75	0.47	b
**OX3**	1.68	0.42	b
**OX4**	5.51	2.22	a
**OX5**	5.91	2.31	a
**OX6**	4.77	1.93	a
**OX7**	6.02	2.59	a
**OX8**	0.35	0.07	cd
**OX9**	0.49	0.10	cd
**Wild-type**	0.35	0.07	cde
**EV1**	0.25	0.05	cde
**EV2**	0.22	0.04	cde
**EV3**	0.21	0.04	cde
**EV4**	0.20	0.03	de
**EV5**	0.28	0.05	cde
**EV6**	0.26	0.05	cde
**EV7**	0.22	0.04	de
**EV8**	0.22	0.04	de
**EV9**	0.19	0.04	de

#### Discriminating dose experiments

Three additional experiments (Experiments A, B, and C) were conducted to confirm results from the dose-response experiment and test for consistent relative resistances when glyphosate was applied to rosettes *vs*. flowering plants (Table 1). These experiments included 6 OX lines, 4–7 EV lines, and the wild-type line, all of which were subjected to discriminating dosages of either 1.0x or 0.5x glyphosate (Table 1). Three of the original OX lines were not used because two had low resistance to glyphosate (labeled OX8, OX9; Table 2), and a third (OX7) produced plants that were severely deformed, with abnormal rosettes and very short internodes at flowering. We also eliminated two randomly chosen EV lines (labelled EV8, EV9; Table 2) because these were deemed unnecessary.

Seeds were germinated in moistened Sun Gro Metro-Mix 360 soil, with one seedling per pot (4.5 or 6 cm square pots), and one pot per line arranged in random positions within trays. The experiments included 15–24 plants per line (i.e., 15–24 trays; Table 1). All trays were irrigated from the bottom every 2 or 3 days and were rotated weekly to account for variation in environmental conditions. Experiments A and B were started in a growth room with 8 hrs of light for one month and then transferred to a greenhouse with supplemental light as above and ambient day lengths (13–15 hrs). For Experiment B, the pots were fertilized twice using 25 mL of nutrient solution (180 ppm 20:10:20; www.jrpeters.com) and then were sub-irrigated with this nutrient solution approximately every 3 weeks. Supplemental light was used to provide 16 hours of daylight. Fertilizer was not used in Experiments A or C. Plants in Experiment C were started over four consecutive days to stagger the timing of leaf samples used for *EPSPS* gene expression (see below).

Plants in Experiments A and B were sprayed at 44 days after planting, prior to flowering, while those in Experiment C were sprayed at 35 days when they had started flowering (Table 1). Glyphosate treatments of 0.5x or 1.0x were applied as described above. Resistance to glyphosate was recorded 21 days after spraying in all experiments. Visual damage scores were “0” for dead plants, “1” for almost dead, “2” for likely to die, “3” for likely to survive, “4” for partly damaged, and “5” for those that were mostly green and had green meristems (center of rosette). This scoring system was deemed to be more efficient and easier to carry out than that used in the dose-response experiment, which involved scores of 1–10. For Experiment A, we also weighed the fresh, above-ground, living biomass of each surviving plant. A one-way ANOVA with Tukey’s multiple range tests was performed to compare above-ground biomass or visual damage scores among the OX, EV, and wild-type lines, using the software IBM SPSS Statistics ver. 19.0 for Windows (SPSS Inc., IBM Company Chicago, IL, USA, 2010).

### Gene expression of *EPSPS*

Quantitative real-time PCR was used to estimate gene expression of *EPSPS* relative to the native *Actin7* gene (e.g., [42]). We compared *EPSPS* expression levels among 6 OX lines, 3 EV lines, and wild-type plants from Experiment C, before glyphosate was applied (Table 1). Because gene expression is likely to vary over time, we staggered the planting and RNA sampling of these lines over four days. Thus, on days 1–4, we planted six plants per line each day, for a total of 24 plants per line. Three plants per line were sampled from rosettes, 28 days after planting, and the other three were sampled at the flowering stage, 35 days after planting, to obtain composite samples (see below). This provided independent estimates of gene expression from different plants at two distinct developmental stages. Then, all 24 plants per line were sprayed with glyphosate at 35 days after planting, as described above.

On each day of sampling, we collected one healthy, young leaf (~1 cm^2^ leaf area, ~75–100 mg) per plant and combined the three leaves per line into a composite sample. Thus, for each line and each stage of development, we obtained four composite samples (replicates), which were frozen immediately in liquid nitrogen and used for RNA extraction. RNA was extracted using RNeasy Plant Mini Kit (Qiagen), quantified using a Nano drop spectrophotometer (ND-1000, Thermo Scientific), and treated by DNase I (Invitrogen). The cDNA was created by a Reverse-transcriptase (RT) reaction using Promega Reverse-Transcription System. For the real-time PCR, all primers were designed using PRIMER 3 (version 0.4.0) (http://bioinfo.ut.ee/primer3-0.4.0/) and synthesized by Eurofins MWG Operon LLC. Primer sequences for *EPSPS* were 5’-TCGTGCTGTAGTTGAAGGATG-3’ (forward) and 5’-GCGGTAAGTGGACGCATT-3’ (reverse); Primer sequence for *Actin*7 were 5’- CAGTGTCTGGATCGGAGGAT-3’ (forward) and 5’- TGAACAATCGATGGACCTGA-3’ (reverse). Three reaction mixes of 25ul for real-time PCR was prepared for each composite sample with iQ^™^ SYBR^®^ Green Supermix from BioRad proportioned according to its user guide. A two-step program on the real-time PCR cycler (Bio-Rad CFX96 Touch^™^) with annealing temperature at 60 C was used with each composite sample. Efficiencies for the *EPSPS* (96.1%) and *Actin7* (104%) primer pairs were determined by slopes of dilution curves with R^2^>0.99. The lack of primer dimers and specificity of each primer was demonstrated through gel analysis, melt curves, and sequencing of products.

The Relative Normalized Quantification of *EPSPS* in each composite sample was calculated based on the modified 2^-ΔΔCT^ method [43,44]. Wild-type samples were used as the control and *EPSPS* and *Actin 7* were the target and reference (ref) genes, respectively. To calculate the relative quantification, the modified equation used is:

$$\frac{{\left(E_{target}+1\right)}^{\Delta Cq_{target}}}{{\left(E_{ref}+1\right)}^{\Delta Cq_{ref}}}$$

where $\Delta Cq_{target}=Cq_{target_{control}}-Cq_{target_{sample}}$, $\Delta Cq_{ref}=Cq_{ref_{control}}-Cq_{ref_{sample}}$, E indicates primer efficiency, and Cq is the quantification cycle. To summarize, the sampling procedure entailed 10 lines x 2 developmental stages x 4 days on which we collected one independent composite sample per line, for a total of 80 composite samples.

The final Relative Normalized Quantification of each OX, EV, or wild-type line at each growth stage (vegetative *vs*. flowering) was calculated by averaging the values of composite samples from different days and weighting the values such that the wild-type line had a value of 1.0. In some lines, no usable data were obtained for one of the four days of sampling, resulting in final sample sizes of 3–4, except for one flowering line, OX1, which had two samples. For each growth stage, the average expression levels of the 6 OX, 3 EV, and the wild-type lines were compared using a two-way ANOVA to account for differences among sampling dates (blocks), with Tukey’s multiple range tests (IMB SPSS Statistics ver. 19.0 for Windows). Although our final sample sizes were lower than planned, differences in gene expression associated with the OX construct were clear and consistent, as shown below.

## Results and discussion

In this study, our main goal was to obtain and characterize independent *Arabidopsis* lines that overproduce *EPSPS* and exhibit enhanced glyphosate resistance. When transgenes are inserted into a plant’s genome, their effectiveness is expected to vary among independent insertion events, such that only a subset of the lines will be useful for further research. Here, all of the EV lines were similar in terms of lacking resistance to glyphosate, as expected, with no visible developmental problems associated with the transgene construct. In contrast, we did observe variation in glyphosate resistance and morphology among the nine OX lines, as discussed below. This variation most likely results from position effects of transgene insertion or insertion of a transgene multimer at the insertion locus, although mutations generated by *Agrobacterium* during the production of T1 plants also may have contributed.

**Fig 2:**
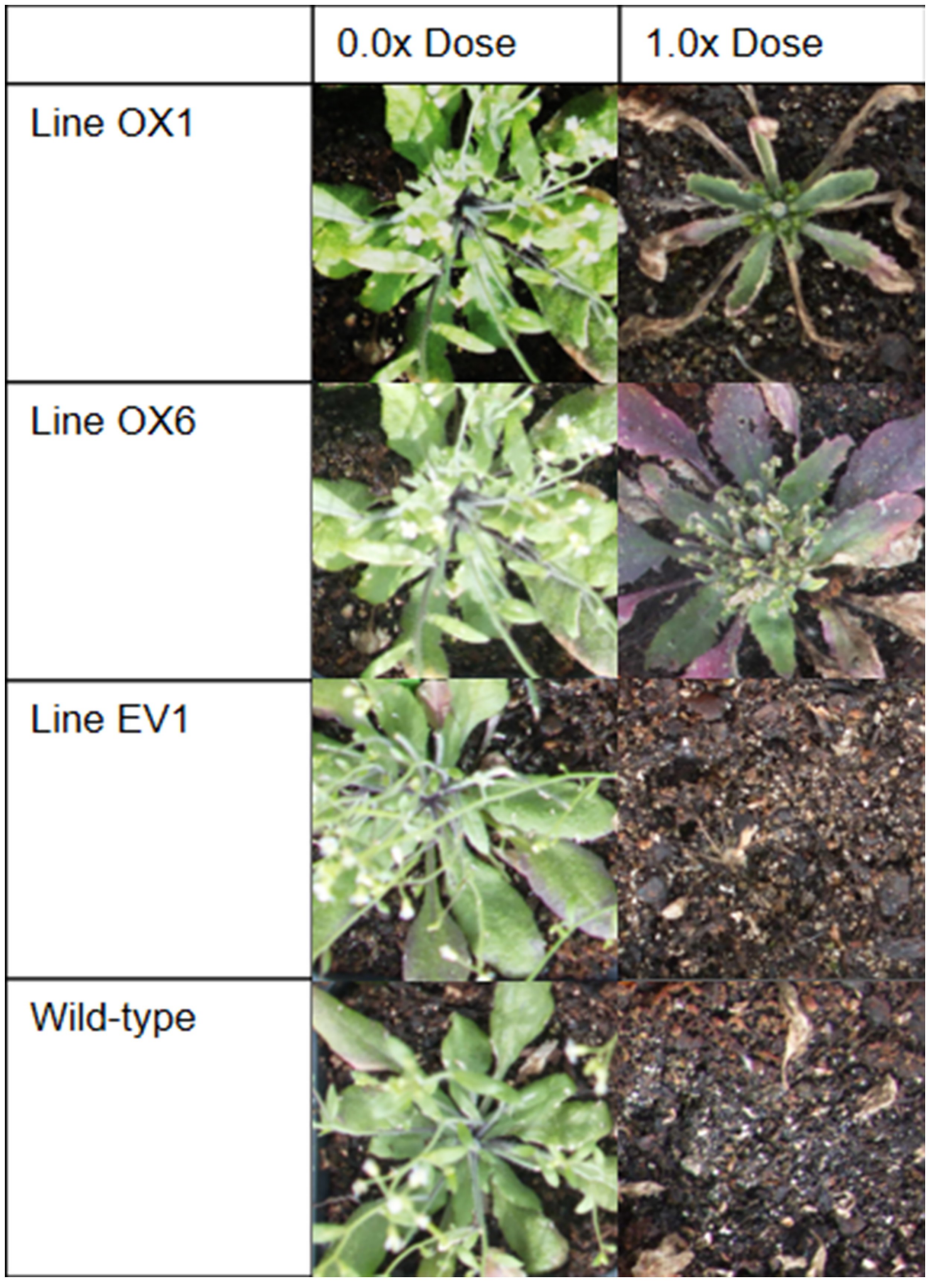
Photos of representative plants from OX, EV, and wild-type *Arabidopsis* lines in Experiment A, showing amount of glyphosate damage from 0.0x dose *vs*. 1.0x dose, 21 days after spraying the vegetative rosettes. Plants with median damage levels in each line are shown.

**Fig 3:**
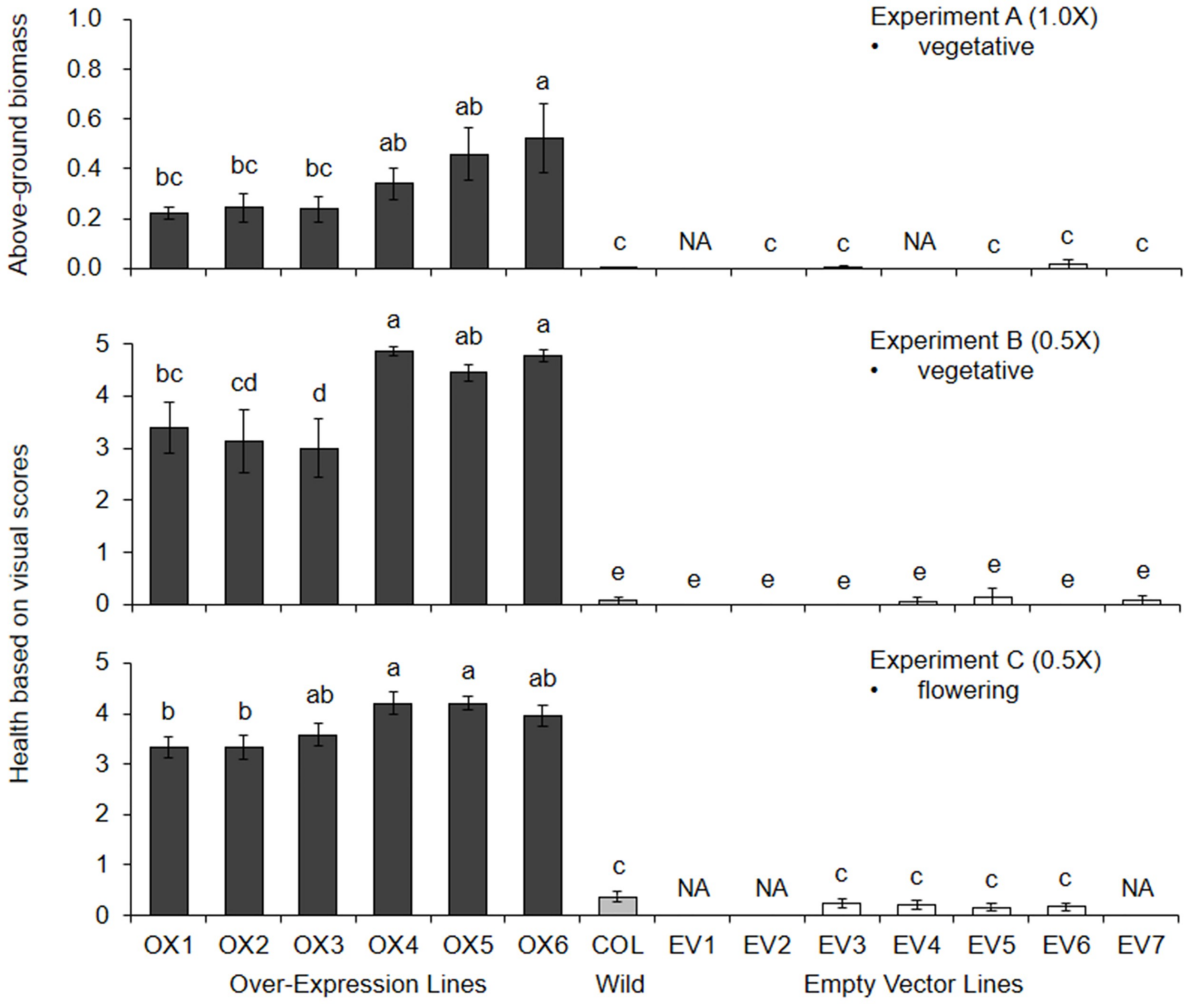
Relative glyphosate resistance of OX, EV, and wild-type *Arabidopsis* lines, showing means ± 1 SE in Experiments A, B, and C at 21 days after spraying with glyphosate (1.0x or 0.5x). Sample sizes and other details are summarized in Table 1. Above-ground biomass (grams fresh weight) is shown for Experiment A. Visual damage scores are based on a scale of 0–5, with 0 for plants that died, and 5 for plants that were mostly green and were developing new leaves. Means that do not share superscripts are significantly different at p<0.05 (Tukey tests). NA indicates “not applicable” for lines that were not used in Experiments A and C.

We found that transgenic insertion of the OX construct conferred greater resistance to glyphosate in 7 of the 9 independent T3 lines, as compared to the EV and wild-type lines (Table 2), representing a relatively high success rate. One of the seven resistant lines, OX7, had a high ED50 (6.02 kg ae/ha), but was eliminated from further study because many plants had deformed rosettes and shortened internodes when flowering. Among the six remaining OX lines, we observed two general levels of glyphosate resistance (Table 2; Figs 2 and 3). Three lines, OX1, OX2, and OX3, had ED50 values ranging from 1.4 kg ae/ha to 1.7 kg ae/ha, while the other three had ED values from 4.8 kg ae/ha to 5.9 kg ae/ha; these differences were significant at p<0.05 (Table 2). Levels of glyphosate resistance in the single-dose experiments were consistent with the dose-response experiment, but differences among the OX lines were less pronounced (Fig 3). In Experiment A, with a 1x glyphosate treatment, surviving biomass of line OX6 was significantly greater than the biomass of OX1, OX2, and OX3 (Fig 3A). Likewise, in Experiment B (0.5x treatment), lines OX4, OX5, and OX6 performed significantly better than OX2 and OX3. In Experiment C (0.5x treatment), OX4 and OX5 were significantly more resistant to glyphosate than OX1 and OX2. In summary, levels of glyphosate resistance among the OX, EV, and wild-type lines were consistent across the four experiments (Table 2; Fig 3).

**Fig 4:**
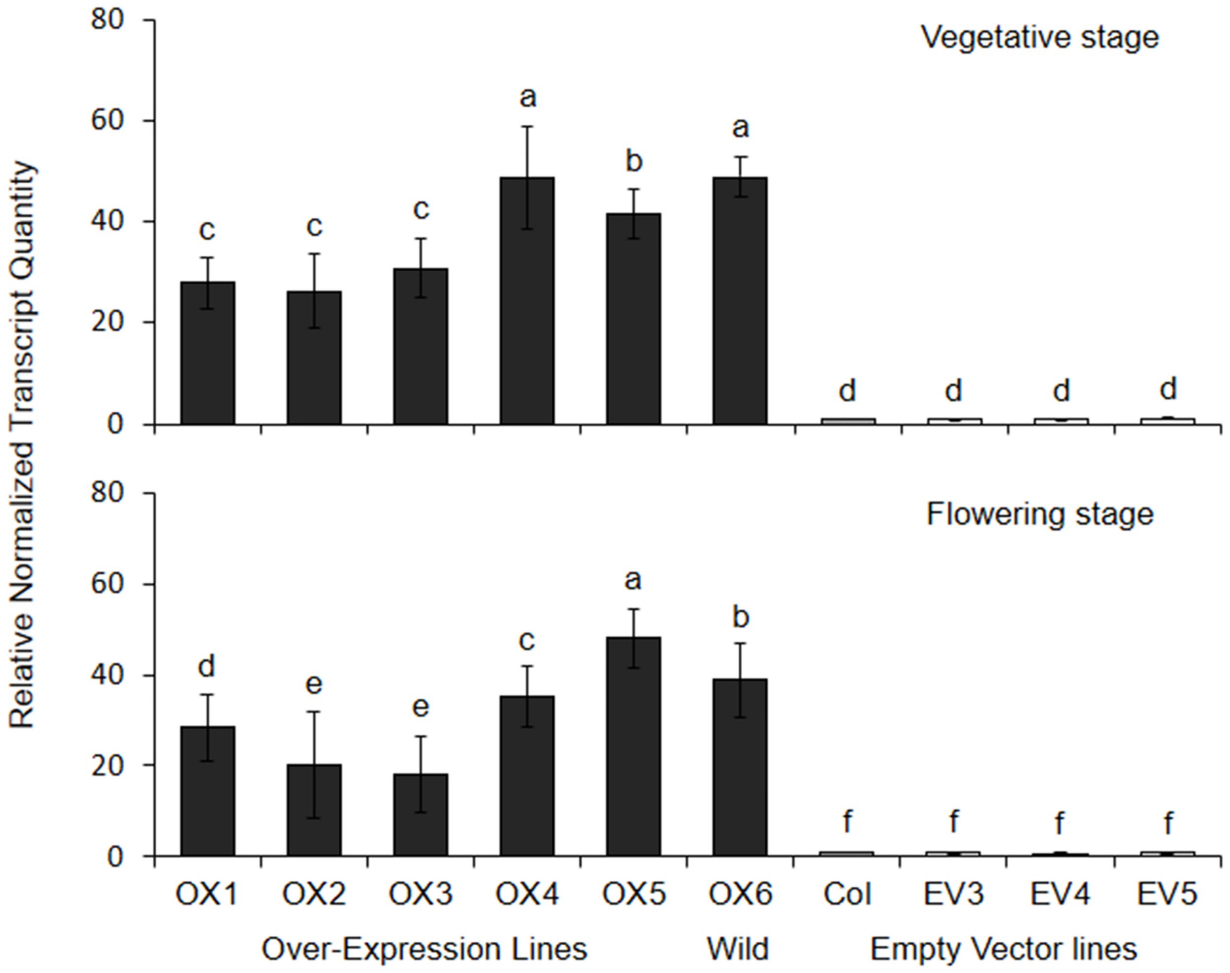
Relative normalized expression of *EPSPS* for OX, EV, and wild-type *Arabidopsis* lines that were used in Experiment C (see Fig 3). Means (N = 3–4) ± 1 SE are shown for non-flowering (top) and flowering plants (bottom; one flowering line, OX1, had N = 2). Means that do not share superscripts are significantly different at p<0.05 (Tukey tests).

We observed enhanced gene expression in the leaves of vegetative rosettes and flowering plants in the OX lines (Fig 4), as expected for leaf samples with a construct driven by the CaMV35S promoter. Vegetative plants in the OX lines had ~26-49-fold greater *EPSPS* gene expression than wild-type controls, while somewhat lower levels of gene expression were found in flowering plants from the OX lines (Fig 4). Among the OX lines, gene expression levels were lower in OX1, OX2, and OX3 compared to OX4, OX5, and OX6, which is consistent with lower glyphosate resistance observed in OX1, OX2, and OX3 (Table 2; Fig 3). Thus, we conclude that greater glyphosate resistance in the OX lines can be attributed to the CaMV35S::*EPSPS* transgene, and variation in levels of over-expression among OX lines were correlated with levels of glyphosate resistance. Some studies have shown that 35S is not expressed strongly in reproductive tissues of other species [45], but we did not test for this effect here.

The long-term goal of our research is to examine other phenotypic effects of overproducing EPSPS in *Arabidopsis* in the absence of glyphosate, including studies of plant growth, competitive ability, and lifetime fitness. These ongoing and future studies are expected to improve our understanding of weed biotypes that have evolved glyphosate resistance by overproducing EPSPS. In addition, we note that The Scotts Company (Marysville, OH, USA) is developing a glyphosate-resistant turf grass, *Poa pratensis*, with over-expression of an *EPSPS* gene from *Arabidopsis thaliana* driven by an ubiquitin promoter from rice, *Oryza sativa* [46,47]. Therefore, insights from transgenic *Arabidopsis* lines that overproduce EPSPS may be relevant to both cultivated and weedy taxa that also overproduce this unique enzyme in the shikimic acid pathway.
